# Succession in the petroleum reservoir microbiome through an oil field production lifecycle

**DOI:** 10.1038/ismej.2017.78

**Published:** 2017-05-19

**Authors:** Adrien Vigneron, Eric B Alsop, Bartholomeus P Lomans, Nikos C Kyrpides, Ian M Head, Nicolas Tsesmetzis

**Affiliations:** 1 School of Civil Engineering and Geosciences, Newcastle University, Newcastle upon Tyne, UK; 2 Shell International Exploration and Production Inc., Houston, TX, USA; 3 DOE Joint Genome Institute, Walnut Creek, CA, USA; 4 Shell Global Solutions International B.V., Rijswijk, The Netherlands

## Abstract

Subsurface petroleum reservoirs are an important component of the deep biosphere where indigenous microorganisms live under extreme conditions and in isolation from the Earth’s surface for millions of years. However, unlike the bulk of the deep biosphere, the petroleum reservoir deep biosphere is subject to extreme anthropogenic perturbation, with the introduction of new electron acceptors, donors and exogenous microbes during oil exploration and production. Despite the fundamental and practical significance of this perturbation, there has never been a systematic evaluation of the ecological changes that occur over the production lifetime of an active offshore petroleum production system. Analysis of the entire Halfdan oil field in the North Sea (32 producing wells in production for 1–15 years) using quantitative PCR, multigenic sequencing, comparative metagenomic and genomic bins reconstruction revealed systematic shifts in microbial community composition and metabolic potential, as well as changing ecological strategies in response to anthropogenic perturbation of the oil field ecosystem, related to length of time in production. The microbial communities were initially dominated by slow growing anaerobes such as members of the *Thermotogales* and *Clostridiales* adapted to living on hydrocarbons and complex refractory organic matter. However, as seawater and nitrate injection (used for secondary oil production) delivered oxidants, the microbial community composition progressively changed to fast growing opportunists such as members of the *Deferribacteres*, *Delta-*, *Epsilon*- and *Gammaproteobacteria*, with energetically more favorable metabolism (for example, nitrate reduction, H_2_S, sulfide and sulfur oxidation). This perturbation has profound consequences for understanding the microbial ecology of the system and is of considerable practical importance as it promotes detrimental processes such as reservoir souring and metal corrosion. These findings provide a new conceptual framework for understanding the petroleum reservoir biosphere and have consequences for developing strategies to manage microbiological problems in the oil industry.

## Introduction

Despite significant efforts to reduce our dependence on energy from fossil fuels, oil, gas and coal still meet around 80% of global energy demand, and International Energy Authority projections indicate that fossil fuels will likely continue to be the dominant sources of energy and transportation fuels for many years (U.S. Energy Information Administration, 2015).

Petroleum reservoirs are an important component of the deep biosphere where autochthonous microorganisms live under extreme temperature and pressure conditions. However, this slow, diffusion-driven deep biosphere environment, which has been relatively stable and isolated over millions of years, is transformed, during oil exploration and production, into an advection-driven system where new electron acceptors, donors and exogenous microbes are introduced to the formerly static environment. Indeed, following primary recovery of oil from a reservoir, where oil and formation water are pushed to the surface by the natural pressure within the reservoir, water injection is used to maintain pressure of the reservoir and achieve higher levels of oil production (secondary recovery). Water injection, however, can have detrimental consequences, notably in offshore facilities when seawater is injected, stimulating deleterious microbial activities such as hydrogen sulfide production (souring), corrosion and reservoir plugging, which in turn lead to increased refining costs and health risks (Youssef *et al.*, 2009). Furthermore, the value and quality of the oil is also strongly influenced by the degree of biodegradation and the presence of metabolic products generated from microbial activity such as sulfur compounds and organic acids. Whether the deleterious microorganisms are indigenous to the reservoir or are also introduced through drilling and water injection remains a subject of debate. Culture-dependent (Magot *et al.*, 2000) and -independent (Orphan *et al.*, 2000; Duncan *et al.*, 2009; Pham *et al.*, 2009; Gieg *et al.*, 2010) analysis of microbial communities in produced fluids, as well as analysis of oil biodegradation (Head *et al.*, 2003) has revealed autochthonous microbial life in oil reservoirs with temperatures below 80 °C (Magot, 2005). Microbial involvement in reservoir processes such as souring and biodegradation compel the oil industry to invest heavily in microbial control strategies such as nitrate injection and biocide addition to limit microbial growth (Voordouw *et al.*, 2009). Despite the practical significance of anthropogenic perturbation of the petroleum reservoir microbiome, there has been no systematic study of how production activities alter microbial communities over the production lifetime of an offshore oil field. Indeed, most studies of microbial communities in oil fields have relied on analysis of onshore low temperature reservoirs (Voordouw *et al.*, 2009), whereas offshore reservoirs have been characterized from relatively few isolated samples (Orphan *et al.*, 2000; Li *et al.*, 2007; Duncan *et al.*, 2009; Gittel *et al.*, 2009). It remains unclear if a single well can be considered representative of an offshore oil reservoir microbiome over time and space. Furthermore, these limited microbial community composition surveys are frequently associated with sparse geochemical data, leaving environmental factors shaping the microbiome of petroleum reservoirs poorly understood. Therefore, to better understand this deep subsurface ecosystem, we wanted to answer several important practical and fundamental questions, which are significant in the context of anthropogenic perturbation of deep subsurface sediment environments: are community composition and metabolic profiles from a single well representative of an entire oil reservoir?; Which environmental factors shape the oil reservoir microbial community composition?; Are there systematic changes in microbial community composition and ecological function driven by oil production practices (for example, secondary oil recovery by seawater injection)?; and what are the likely drivers of these shifts? The answers to these questions have important implications not only for petroleum production but for any process where geological resources from deep subsurface environments are exploited. They also offer insights on fundamental aspects of the deep biosphere such as the effects of changes in the mass transfer regimen in slow diffusion-driven deep subsurface sediment ecosystems, the effects of overcoming limitations in electron acceptor and donor availability and the ability of exogenously introduced organisms to establish themselves in perturbed deep subsurface environments.

The Halfdan oil field, Danish North Sea, is situated 2.1 km below the seafloor. The average temperature is 80 °C and the field harbors two oil accumulations, each with distinct formation waters (that is, undisturbed water naturally associated with the geological formation; formation water A (FWA) in the North and formation water B (FWB) in the South, East and West of the field). In addition, the field includes a gas reservoir (Ekofisk) (Larsen *et al.*, 2004). Oil and gas recovery in the field is based on Fracture Aligned Sweep Technology, where long horizontal wells are arranged in a parallel pattern of alternate production and seawater injection wells (Albrechtsen *et al.*, 2001) (Supplementary Figure S1). This technology generates a continuous water front along the length of the oil field, driving the oil toward the production wells. During secondary oil production in the Halfdan oil field, seawater is deoxygenated and continuously enriched with 100–150 mg l^−1^ of nitrate before injection as a strategy for the prevention of souring and metal corrosion. Indeed, continuous nitrate injection was demonstrated to efficiently limit corrosion problems in the early years of production in the Halfdan oil field (Larsen *et al.*, 2004).

Analysis of an unrivaled sample set representing different production stages of the Halfdan oil field, ranging from early primary, through to late secondary oil production, has allowed us to assess emergent microbial community properties across the 32 producing wells over the 15 year operational history of the field. In combination with physicochemical data available for each of the wells, this study provided a unique opportunity to understand not only the interactions between reservoir conditions and microbial community composition, but also the interplay among the measured physicochemical parameters.

## Materials and methods

### Sampling and DNA extraction

**Figure 1:**
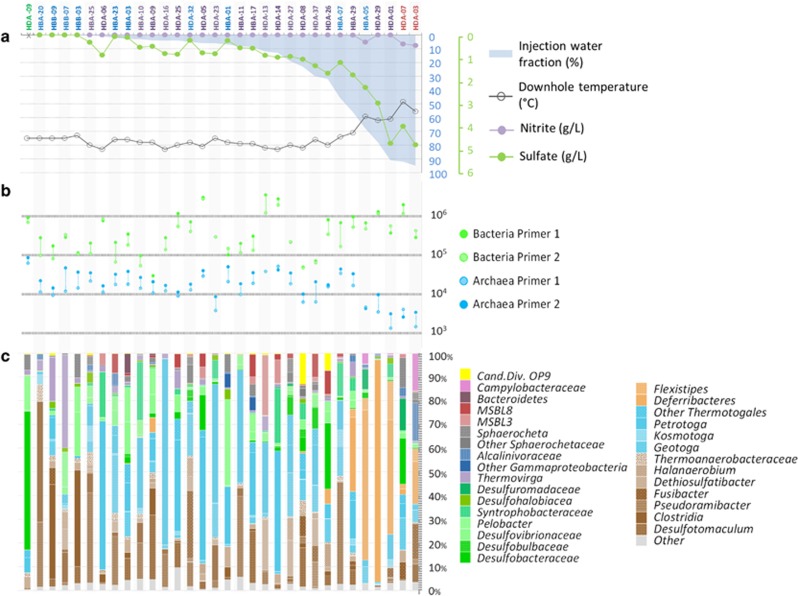
(**a**) Downhole temperature (black circle), proportion of injected seawater (blue shading), sulfate (green dots) and nitrite (purple dots) concentrations in the produced water from the different wells (sulfate and nitrite concentrations were not available for HDA-09 sample). The blue scale relates to temperature and the proportion of injected water. The green scale is for nitrite and sulfate concentration. Color code of the producing wells ID indicates the corresponding formation water type (blue text: FWA; purple text: FWB; green text: gas-containing formation; red text: mixed source). (**b**) Bacterial (green) and archaeal (blue) 16S rRNA gene abundance per milliliter of produced fluid. Quantification was done with two different primer sets for each superphylum for an optimal estimation of the abundance. (**c**) Taxonomic affiliation of the bacterial 16S rRNA genes amplified from all produced fluids (average of the two experimental duplicates). Brown colors indicate members of the *Firmicutes*; green, *Deltaproteobacteria*; dark blue, G*ammaproteobacteria*; light blue, *Thermotogales* and orange, *Deferribacteraceae*. Taxonomic affiliation of the archaeal 16S rRNA gene amplicons is also provided in Supplementary Figure S1 and S2.

Produced fluids (that is, liquid recovered from the producing well) from 32 producing wells, drilled at different times since 1999 and covering the entire Halfdan oil field (~60 km^2^) were collected in December 2014 (Supplementary Table S1), each representing a different stage of the production lifetime of the field. Sampling for microbial community composition analysis and geochemical analysis was conducted simultaneously. Geochemical analysis for each of the well samples was carried out by the platform operator and AquaTeam COWI and is reported in Supplementary Table S1 and Figure 1a. Downhole nitrate concentrations measured in 2013 are reported in Supplementary Table S1. The proportion of injected seawater in the produced fluids was estimated based on the concentration of conservative ions using the Ion Tracking method. This method compares the ion concentrations from produced fluids with both formation water and seawater ion concentrations to evaluate the mixing of the fluids and incorporates geochemical modeling to account for changes in water composition because of mineral precipitation or dissolution (Ishkov *et al.*, 2009). For microbial community composition analysis, an average of 85±35 ml of produced fluids was collected at each producing well and directly filtered on Sterivex filters (EMD Millipore, Darmstadt, Germany) or 0.22 μm membrane filters (EMD Millipore). Filters were then stored at 4 °C in RNAlater (Life Technologies, Carlsbad, CA, USA) until DNA extractions were conducted. Nucleic acids were extracted in a sterile hood using a FastDNA Spin Kit for Soil (MP Biomedicals, Santa Ana, CA, USA) with modifications as described in Supplementary Material. Nucleic acids were dissolved in 50 μl of RT-PCR grade water (Ambion, Life Technologies) resulting in contaminant-free DNA at a concentration allowing metagenome shotgun sequencing without further purification, concentration or whole-genome amplification steps.

### Quantitative PCR

To ensure an accurate estimation of the microbial abundance, 16S ribosomal RNA (rRNA) genes from *Bacteria* and *Archaea* were quantified by real-time quantitative PCR (qPCR) with four different primer sets (Supplementary Table S2). Additional qPCR experiments, targeting 16S rRNA genes from *Epsilonproteobacteria*, *Deferribacteres*/*Flexistipes*, *Achaeoglobales* and *Thermococcales* lineages were also carried out using specific primer sets (Supplementary Table S2). Amplification reactions were performed in triplicate as detailed in the Supplementary Methods. Varying amounts of DNA (1, 0.5 and 0.1 ng) were used as qPCR template in the triplicate analyses to identify potential inhibition of the reaction. qPCR results from the duplicate samples were averaged and abundances were expressed in terms of 16S rRNA gene copies per ml of produced water (Figure 1b).

### Library preparation and sequencing

16S rRNA genes were amplified in duplicate from two independent DNA extracts (* and ¤) as presented in the Supplementary Material. These were analyzed as separate replicates. In addition, *mcrA* and *dsrAB* genes were also amplified in triplicate from a single DNA extract per sample as described in Supplementary Material. PCR amplicon replicates were pooled and gel-purified on an agarose gel before being indexed using a Nextera XT Kit (Illumina, San Diego, CA, USA) according to the manufacturer’s recommendations. Indexed amplicons were diluted to 4 nM then pooled together. After final dilution, 4 pM libraries containing 5% phiX V3 control DNA (Illumina) were loaded on Illumina MiSeq V3 cartridges (Illumina) for sequencing.

Metagenomes for eight produced fluid samples, selected based on their contrasted community composition, were constructed using a Nextera XT library Kit (Illumina) according to the manufacturer’s recommendations with 1 ng of genomic DNA. An equal volume (2.5 μl) of DNA replicate (*) and (¤) were pooled and used as DNA template. Fragmentation and indexing were checked using a High Sensitivity DNA chip on an Agilent Bioanalyzer 2100 (Agilent Technologies, Santa Clara, CA, USA) then libraries were diluted to 14.3 pM and sequenced using Illumina Miseq V3 kit (Illumina).

### Sequencing data analysis

MiSeq runs were demultiplexed, then adapter and barcode sequences were trimmed on instrument using Illumina’s MiSeq Reporter software. For amplicon sequencing, FASTQ files were downloaded from the instrument. Quality filtering, paired-end joining and operational taxonomic unit taxonomic assignment were carried out using QIIME (version 1.9) (Caporaso *et al.*, 2010) with the 16S rRNA Silva database (version 1.19) (Quast *et al.*, 2013), a publicly available *dsrAB* gene database (Muller *et al.*, 2015) and an in-house *mcrA* gene database, as detailed in the Supplementary Material. Sequences are available from the NCBI under Bioproject number PRJNA348365. For metagenome sequencing, quality filtering using Sickle (Joshi and Fass, 2011), paired-end joining, and assembly using MEGAHIT (Li *et al.*, 2015) were performed as described in Supplementary Methods. Contigs were uploaded to the IMG/M analysis pipeline (Markowitz *et al.*, 2008) for gene calling and functional annotation. Metagenomes are available from IMG under accession numbers: 3300005062, 3300005067, 3300005068, 3300005078, 3300005081, 3300005082, 3300005086 and 3300005101. To generate longer contigs and allow for the highest likelihood of producing near-complete genomic bins, the reads from all eight metagenomes were concatenated, normalized, then assembled using an Iterative De Bruijn graph de novo Assembler for Uneven sequencing Depth data (IDBA-UD) (Peng *et al.*, 2012) as detailed in Supplementary Methods. Resulting bins were analyzed for quality and completeness against single copy genes using CheckM (Parks *et al.*, 2015). Bins with completeness above 50% and contamination level under 3% were selected for further analysis. Metabolic pathways were considered as present in the assembly if >75% of the pathway genes were detected using MetaCyc (Caspi *et al.*, 2010) and the KEGG pathway mapping tool. In addition, key genes for functional pathways were also manually screened to validate the presence of the pathway in the genomic bins. For *mcrA*, *dsrAB*, *pmoA*, *assA*/*bssA*/*masD*, *mtrAB*/*omcAB* genes analysis, contigs were compared against publicly available databases using USEARCH (Edgar, 2010).

### Statistical analysis

Statistical analyses of the data set (Correlation tests principal components analysis (PCA), similarity percentages breakdown procedure (SIMPER), analysis of similarity tests (ANOSIM)) were carried out according to recommendations of the Guide to Statistical Analysis in Microbial Ecology (Buttigieg and Ramette, 2015), using R with ade4TKgui and Vegan packages (Dixon, 2003). Redundancy analysis (RDA) was carried out in CANOCO and visualized in CANODRAW (Lepš and Šmilauer, 2003).

## Results

### Geochemical characteristics of the Halfdan oil field

Composition and geochemical characteristics of the produced fluids from the 32 sampled wells were analyzed (Figure 1, Supplementary Table S1). Two distinct formation waters (that is, undisturbed water naturally associated with the geological formation) are present in the Halfdan oil field (Supplementary Figure S1). In the northern accumulation, formation water (FWA) had a moderate salinity (38 p.p.t.), as well as low concentrations of metal ions (for example, Mg^2+^, K^+^) and sulfate (Supplementary Table S1). By contrast, in the second oil accumulation of the field, formation water (FWB) had a higher salinity (FWB: 72 p.p.t.), as well as relatively higher concentrations of metal ions (for example, Mg^2+^, K^+^) and sulfate (Supplementary Table S1), probably because of the influence of a salt dome in the southern and western boundaries of the field (Albrechtsen *et al.*, 2001) (Supplementary Figure S1).

**Figure 2:**
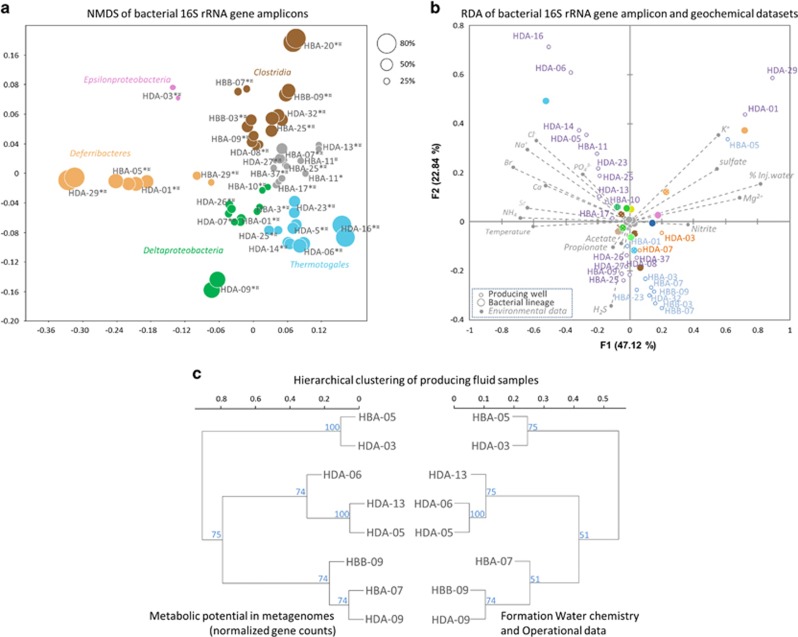
(**a**) Non-metric multidimensional scaling (NMDS) of the bacterial 16S rRNA gene amplicons data set (stress=0.0002). Size of the dot represents relative proportion of the dominant lineage in each sample. Color code is the same as in Figure 1. Gray dots indicate a mixed microbial community without a particular predominant bacterial lineage. (**b**) RDA of bacterial 16S rRNA gene amplicons data set (large dots color coded as in Figure 1) with geochemical parameters (gray vectors, see Supplementary Table S1 for details of all geochemical data). The RDA plot illustrates the correlation between community composition and geochemistry in produced fluid samples (gray dots). The color of the produced water sample dot border and sample name indicate the formation water type (blue: FWA; purple: FWB; orange: mix of FWA and FWB). Samples HDA-09 and HBA-20 were not included in the analysis because of limited water chemistry data. (**c**) Hierarchical clustering of produced fluid samples based on (1) physicochemical data (left-hand dendrogram) and (2) metabolic potential (relative abundance of 2.25 × 10^5^ normalized Kegg Orthologies (KOs); right-hand dendrogram). HDA-03 and HBA-05 represent samples with a high seawater fraction (>68%). HBA-07 represent samples with moderate seawater fraction (<50%) HDA-05 and HDA-13 represent samples with low seawater (<12%) and FWB, and HBB-09 represent samples with low seawater and FWA. HDA-09 represents the gas-containing formation. Blue numbers represent percentage agreement at the node over 10 000 bootstrapped replicates.

Different proportions (0–95%) of injected seawater in produced fluids were observed depending on the age of the wells and the progression of the water front through the oil reservoir (Figure 1). Wells were clearly delineated based on the proportion of seawater reaching the producing well (% Inj. Water) and on the produced water composition, which is strongly influenced by the formation water type (Figure 2b—different colored water sample labels correspond to different formation water types). Chemical data follow this pattern with parameters associated with injected seawater (magnesium, potassium, nitrate, nitrite and sulfate) clearly separating from characteristics related to formation water (including volatile fatty acids, ammonium, sodium and chloride) (Figure 2b). Lower *in situ* temperature (down to 42 °C), higher sulfate (up to 4787 mg l^–1^), nitrate (up to 43 mg l^–1^) and nitrite (up to 7.6 mg l^–1^) concentrations were detected in cases of seawater breakthrough, whereas higher temperature (80 °C), higher ammonium (up to 52 mg l^–1^) and volatile fatty acids (up to 22-fold more acetate, propionate and butyrate, *P*<0.01, Supplementary Table S1) were measured in wells with a lower (<50%) proportion of injected seawater.

### Microbial community composition

DNA extraction from the 32 produced fluid samples was performed in duplicate and followed by PCR amplification, amplicon sequencing and quantitative PCR of several genes (16S rRNA, *mcrA* and *dsrAB*) to determine the microbial community composition and abundance in all produced fluids. In addition, shotgun metagenome sequencing of eight samples, selected based on their community composition and produced fluid geochemistry was performed. 16S rRNA genes recovered from the metagenome data were extracted and analyzed to confirm the microbial community composition observed by amplicon sequencing.

A microbial (*Bacteria* plus *Archaea*) community of comparable cell density to seawater was detected in the produced fluids (average: 5.87 × 10^5^ 16S rRNA genes ml^−1^; min: 3 × 10^4^ genes ml^−1^; max: 3.34 × 10^6^ genes ml^−1^) (Figure 1b). No significant differences in taxonomic affiliation of the 16S rRNA amplicons were observed (ANOSIM, *P*>0.4) between each of the replicates (Figure 2a). The microbial community composition determined from 16S rRNA gene amplicons and 16S rRNA gene sequences recovered from metagenomes were congruent (Pearson correlation coefficient *r*>0.8 and *P*<0.03 for all eight samples, Supplementary Figures S3 and S4).

*Archaea* represented 1–38% of the microbial community composition based on both qPCR and metagenomic data (Pearson correlation coefficient *r*=0.86, *P*=0.02). Although archaeal abundance was significantly lower in samples with seawater breakthrough (~5.5 × 10^3^ 16S rRNA genes ml^−1^;*T*-test, *P*=0.02), archaeal communities were similar in all producing wells and were dominated by thermophiles from the families *Archaeoglobales* and *Thermococcales* (Supplementary Figure S2). Methanogenic lineages (mainly *Methermicoccales*, *Methanolobales* and *Methanomicrobiales* lineages) were infrequently detected by 16S rRNA and *mcrA* gene sequencing (Supplementary Figures S2 and S8).

By contrast, different bacterial communities (62–99% of the microbes) were detected in the produced fluids (Figure 1). These were dominated either by members of the Thermotogales (explaining 20.7% of the dissimilarity between samples; SIMPER analysis), *Proteobacteria* (20.53% of the dissimilarity; SIMPER analysis), *Clostridia* (22.21% of the dissimilarity; SIMPER analysis) or *Deferribacteres* (16.65% of the dissimilarity; SIMPER analysis) (Figures 1 and 2a). Sulfate-reducing lineages (mainly Archaeoglobales and *Deltaproteobacteria*) were detected in all producing wells by 16S rRNA and *dsrAB* genes sequencing (Supplementary Figure 7).

RDA, highlighting correlations between environmental parameters and community composition in the samples, indicated a strong influence of the proportion of injected seawater (% Inj. Water) on bacterial community composition. Strong correlation was detected between the relative proportion of *Deferribacteres/Flexistipes* lineages (orange dots in Figure 2b) detected by 16S rRNA gene sequencing and the proportion of injected seawater and related environmental parameters (potassium, magnesium, nitrite and sulfate concentrations) measured in samples HBA-29, HDA-29, HDA-01 and HBA-05. In those producing wells with substantial seawater breakthrough (56–91% injection water fraction), the representation of sequences from members of the *Deferribacteres* was significantly greater as determined by both 16S rRNA gene (*T*-test, *P*=0.001) and metagenome (*T*-test, *P*=0.05) analysis. In addition, the *Bacteria* to *Archaea* ratio increased by more than sixfold (*T*-test, *P*=0.02) in those wells, mainly because of a significantly lower archaeal abundance (Figure 1).

Furthermore, RDA analysis indicated a relationship between relative abundance of *Epsilionproteobacteria* (pink dot in Figure 2b) and *Gammaproteobacteria* (dark blue dot in Figure 2b) detected by 16S rRNA gene sequencing, and nitrite concentration measured in HDA-03. The proportion of reads from *Epsilonproteobacteria* and to a lesser extent *Gammaproteobacteria* was greater in 16S rRNA gene (*T*-test, *P*<0.01) and metagenome data sets (for *Epsilonproteobacteria P*<0.005; *Gammaproteobacteria P*<0.04; Supplementary Figure S3) for the producing wells HDA-03 and HDA-07, which exhibited a very high fraction of injection water (that is, >91%). Consistently, qPCR assays, using specific *Epsilonproteobacteria* primers, indicated a higher abundance of *Epsilonproteobacteria* in samples from wells exhibiting the highest proportion of seawater breakthrough. (Supplementary Figure S5).

RDA also revealed a strong influence of the formation water composition on bacterial community composition. Producing wells were clustered according to their formation water (indicated by the color of the outline of the sample dots and names in Figure 2b) as a probable consequence of the formation water on the produced fluid composition. This segregation was more pronounced in wells where produced fluids contained <10% seawater (*n*=17, Figure 1) and where the influence of seawater solutes on produced fluid composition was negligible. RDA analysis indicated a strong correlation between the proportion of *Petrotoga* 16S rRNA genes and produced fluid components measured in samples from FWB and a low proportion of seawater (dots with purple outline in Figure 2b; for example, HDA-16, HDA-06, HDA-14, HDA-05, HBA-11 etc). By contrast, the relative proportion of 16S rRNA genes related to Clostridia correlated with the low salinity and low ion concentrations measured in samples from FWA (dots with blue outline in Figure 2b; for example, HBB-03, HBB-07, HBB-09, HDA-32, HBA-07 etc). Considering only wells where produced fluids contained <10% seawater (*n*=17), bacterial communities were significantly different (ANOSIM *r*=0.51, *P*<0.005) depending on the formation water composition (Supplementary Figure S6). In wells producing from the northern oil accumulation with formation water FWA, members of the *Clostridia* (13±16% s.d.) (*Firmicutes*), *Pelobacter* (11±10% s.d.) (*Deltaproteobacteria*) and *Thermovirga* (8±13% s.d.) (*Synergistes*) were predominant. By contrast, in samples from the second oil accumulation with formation water FWB, and where produced fluids, comprised <10% seawater, the bacterial communities were dominated by members of the genera *Petrotoga* (36±22% s.d.) (*Thermotogae*) and *Desulfotomaculum* (16±13% s.d.) (*Firmicutes*). However, wells HBA-09 and HBA-25 from FWB seem to diverge from this general pattern with a community composition more similar to communities observed in produced fluids from FWA. These discrepancies may be because of unrecognized connectivity between these FWB wells and regions of the reservoir associated with FWA.

### Microbial metabolic potential

**Figure 3:**
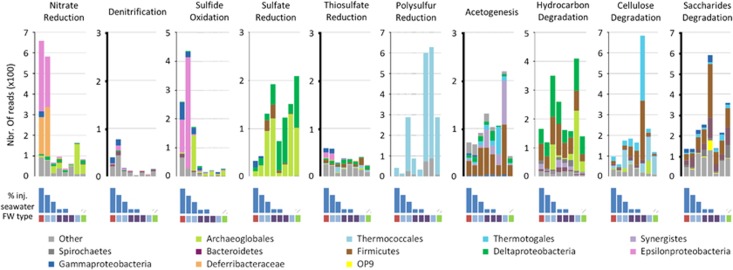
Relative proportion (x100) of selected genes in the normalized metagenomes with IMG/M taxonomic affiliation of the reads. Nitrate reduction gene: *narG*, Denitrification: *nirK*, Sulfide oxidation: *sqr*, Sulfate reduction: *dsrAB*, Thiosulfate reduction: *phsA*, Polysulfur reduction: *hydG*, Acetogenesis: *fhs*, Hydrocarbon degradation: *bssA*, Cellulose degradation: cellulase, Monosaccharide degradation: glycosylase. From the right to the left: HDA-03, HBA-05, HBA-07, HDA-13, HDA-05, HDA-06, HBB-09 and HDA-09. Blue histograms show seawater fraction on the produced fluid (not available for HDA-09) and color code below indicates the formation water type (color code is the same as in Figure 1: blue: FWA; purple: FWB; green: gas formation; red: mixed).

Shotgun metagenomes from eight contrasting samples were sequenced to explore the metabolic potential of the oil field microorganisms. Different metabolic profiles were apparent in the different samples (Figures 2c and 3). Hierarchical clustering (Euclidean distances) of the overall metabolic potential of the wells (relative abundance of 2.25 × 10^5^ normalized functional genes) was generally congruent with the clustering of the wells based on geochemical and operational data (Figure 2c). Samples HBA-05 and HDA-03, presenting apparent seawater breakthrough (% Inj. Seawater >50%) were also clustered together based on their metabolic potential. Similarly, samples HDA-06, HDA-13 and HDA-05, representing the samples from FWB with a low seawater proportion shared a similar metabolic potential. However, samples from gas-producing well HDA-09, clustered together with HBB-09 and HBA-07 in the analyses based on geochemical data or metagenome data (Figures 2b and c). However, the microbial community present in HDA-09 was quite distinct from the communities in HBB-09 and HBA-07 (Figure 2a). The hierarchical clustering of the metabolic potential does not take into account the taxonomic affiliation of the genes but only the relative numbers of the genes regardless of the community composition. Therefore, the similarity of HDA-09, HBB-09 and HBA-07 may result from different communities with similar metabolic potential. Nevertheless, this apparent anomaly clustering could also be a consequence of the limited number of samples included in the analyses. HBB-09, HBA-07 and HDA-09 being the unique representative of their respective environmental conditions, this clustering may be by default, although supported by bootstrap analysis.

In producing wells with a high injection water fraction (68% and above), there was evidence for considerable potential for nitrate reduction by members of the *Epsilonproteobacteria* and *Deferribacteraceae* (Figure 3). Similarly, potential for sulfide oxidation, and to a lesser extent, denitrification and thiosulfate reduction by *Epsilon*- and *Gammaproteobacteria* was identified in seawater-flooded wells. By contrast, a lower potential for sulfate and polysulfur reduction was detected in these wells than in wells with lower injection water fraction. Considerable potential for degradation of hydrocarbons (mainly by members of the *Deltaproteobacteria*, *Firmicutes*, *Archaeoglobales* and *Synergistes* lineages), and (poly)-saccharides (mainly by members of the *Firmicutes*, *Thermotogales* and Candidate division OP9 lineages), and for acetogenesis (*Firmicutes*, *Synergistes, Thermotogales* and *Deltaproteobacteria*) was apparent in wells with a low proportion (below 10%) of injected seawater (Figure 3).

### Genomes assembled from community metagenomes

**Figure 4:**
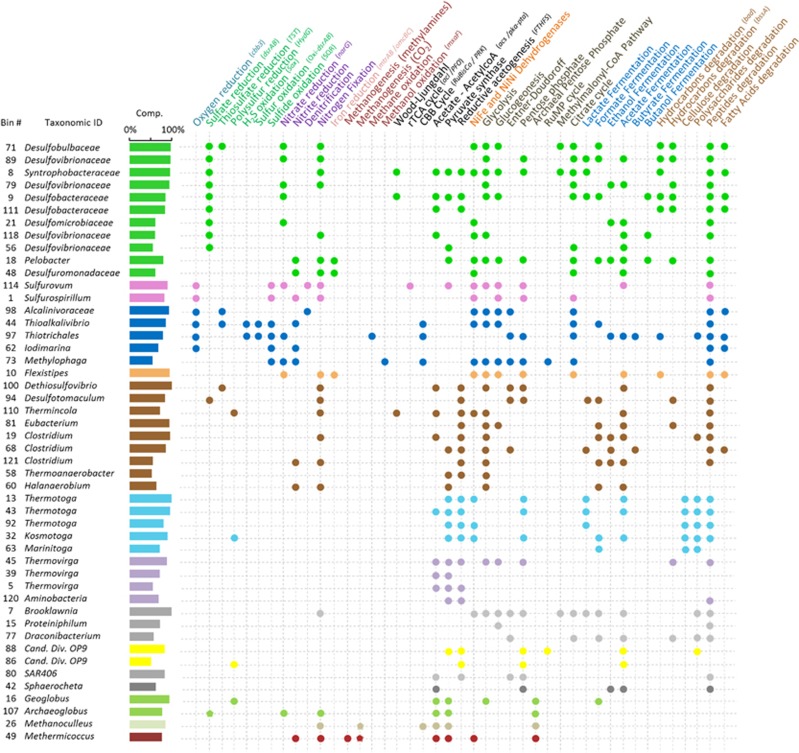
Metabolic pathways identified in reconstructed genomic bins with completeness above 50% and contamination level <3% (CheckM analysis). Taxonomic identification of the bins was based on full 16S rRNA sequence when present and other phylogenetically informative marker genes when no 16S rRNA gene was present. The color code corresponds to the taxonomic affiliation in Figure 1. Pathways were considered as present when >75% of the genes involved in the pathway were detected. Identification of the pathways was carried out with MetaCyc and the KEGG pathway mapping tool. Results were manually checked. Results for specific pathways (iron reduction, sulfate reduction, methanogenesis and hydrocarbon degradation with *bssA* gene) were also confirmed by blast of the bins against in-house databases for these specific genes. Presence of rTCA and the Calvin Benson Bassham cycles was estimated by detection of key genes (*acl* and PFO genes for rTCA cycle and RuBisco and PKR genes for the CBB cycle). *cbb3*, cytochrome C oxidase; *dsrAB*, dissimilatory sulfite reductase; TST, thiosulfate sulfurtransferase; *HydG*, sulfhydrogenase; *sqr*, sulfide:quinone reductase; *narG*, nitrate reductase; *mtrAB*, decaheme c-Cytochromes; *omcBC*, outer-membrane decaheme c-Cytochromes; *mxaF*, methanol dehydrogenase; *acl*, formate C-acetyltransferase; PFO, pyruvate:ferredoxin oxidoreductase; PRK, phosphoribulokinase; *acs*, acetyl-CoA synthetase; *pka*, phosphate acetyltransferase; *fthfs*, formyltetrahydrofolate synthetase; *bad*, benzoate-CoA ligase; *bssA*, benzylsuccinate synthase.

Genomic bins corresponding to representatives from a number of microbial lineages were assembled from the combined metagenomic data sets (Figure 4). Metabolic capabilities were inferred from these genomic bins (completeness above 50% and contamination level <3%). The recovered genomes exhibited contrasting metabolic potentials, which reflected their taxonomic affiliation.

Overall, the reconstructed genomic bins suggested a predominance of heterotrophy. The bacterial bins revealed a large repertoire of pathways for organic matter metabolism (peptide, fatty acid, polysaccharide degradation) including fermentation (acetate, lactate, formate and hydrogen production) (Figure 4). Hydrocarbon degradation genes, such as homologs of the benzylsuccinate synthase gene (*bssA*) for activation of alkyl-substituted aromatic hydrocarbons, were detected in most of the deltaproteobacterial genomic bins, in two bins from *Clostridiales* (*Thermicola* and *Eubacterium*), in *Draconibacterium* and in the most complete bin from a *Thermovirga sp.*. Gene clusters encoding the pathway for anaerobic degradation of monoaromatic compounds (*bad* genes) were identified in *Desulfobacteraceae*, *Thioalkalivibrio* (*Gammaproteobacteria*) and *Flexistipes* (*Deferribacteraceae*) bins.

Various high-energy yielding metabolic pathways were identified. The capacity for aerobic respiration at low oxygen concentration (high-affinity cbb3 cytochrome oxidase) was found in *Epsilon*- and *Gammaproteobacteria* bins. Sulfate reduction potential (*aprA*, *dsrAB*) was identified in *Archaeoglobus*, *Desulfotomaculum* (*Firmicutes*) and *Deltaproteobacteria* genomic bins, but these genes were not found in bins from members of the Desulfuromonadales (*Desulfuromonadaceae* and *Pelobacter*). Genes involved in thiosulfate reduction were detected in *Desulfobulbaceae*, *Dethiosulfovibrio* (*Firmicutes*) and in *Alcanivoracaceae* and *Thioalkalivibrio* (*Gammaproteobacteria*) bins. Polysulfur reduction potential was detected in bins affiliated to *Eubacterium*, *Kosmotoga*, Candidate division OP9 and *Geoglobus* lineages. Hydrogen sulfide- (Sox) and sulfur- (Oxi-dsr) oxidation genes were identified in *Thioalkalivibrio* and *Thiotrichales* genomes and sulfide:quinone reductase gene encoding SQR for the oxidation of sulfide to polysulfide were detected in both *Epsilon-* (*Sulfurovum* and *Sulfurospirillum*) and *Gammaproteobacteria (Thioalkalivibrio, Thiotrichales, Iodimarina* and *Methylophaga*) genomics bins. Genes for nitrate or nitrite reduction were identified in *Delta*- *Epsilon*- and *Gammaproteobacteria* bins. Nitrate reduction potential was also detected in a *Flexistipes* (*Deferribacteraceae*) bin. The capacity for iron reduction via extracellular multiheme cytochromes (*mtrA/omcB*) was also detected in *Flexistipes* as well as in *Pelobacter* and *Desulfuromonadaceae* genomic bins. Methane (*pmoA*) or methanol (*mxaF*) oxidation genes were detected in gammaproteobacterial *Thiotrichales* and *Methylophaga* bins respectively, whereas an autotrophic methanogenesis pathway was identified in *Methanoculleus* and *Methermicoccus* bins (Figure 4).

## Discussion

There have been very few studies of the geochemistry and microbial communities across an entire oil field. A series of studies of the onshore, Medicine Hat Glauconitic C (MHGC) field provide a notable exception (Voordouw *et al.*, 2009; Folarin, *et al.*, 2013). The present study of the Halfdan field is, however, unique in that it focused on an offshore oil field with a far larger areal extent than MHGC and included sampling of more than double the number of wells. The injection fluid for secondary production in Halfdan was nitrate-amended seawater whereas in MHGC it was either produced water or a mix of produced water and effluent from a municipal sewage works (Voordouw *et al.*, 2009). Moreover, in the present study produced water samples provided a systematic representation of parts of the field that had been under production for different periods of time over a 15 year period.

### Multiple microbiomes in a single oil field

Although we do not present a conventional longitudinal study, in that a single well was not sampled over time to assess succession in the microbial communities, we have used the sampling of wells from the same field that have been in production for different periods, as a proxy for time. Given the sampling constraints in an operating oil field, and that the provenance of the produced waters from the different formations in the field is relatively well constrained we consider this to be a pragmatic and defensible approach. Quantitative PCR, sequencing of single genes (16S rRNA, *mcrA* and *dsrAB* genes) and metagenomic analysis of the produced fluids from 32 wells in the Halfdan oil field revealed considerable variation in terms of microbial community composition, abundance and *in situ* metabolic potential. Our data clearly demonstrated that contrasting microbiomes identified in different wells from a single oil field were related to the production history of the wells and hence associated geochemistry. Depending on the proportion of injected seawater in producing fluids, reflecting the progression of oil through the reservoir and the different periods in the production lifecycle of the oil field, microbial community composition was dominated either by members of the *Thermotogales*, *Clostridia*, *Deferribacteres* or *Proteobacteria.* The metabolic capacities of these microbial communities were respectively centered either on degradation and fermentation of detrital organic matter (cellulose and other complex glycans such as laminarin and fucose), acetogenesis and aromatic hydrocarbon degradation, or, nitrate reduction and oxidative processes such as hydrogen sulfide, sulfide or sulfur oxidation, as demonstrated by metagenomic mining and genomic bin reconstruction. An important consequence of such differences in community composition and function from a single oil field is that community composition or metagenomic reconstruction from a single well, are not representative of the entire reservoir but rather relate to a specific stage in an oil field production cycle. This finding has serious implications for microbial monitoring and control practices for field operators.

### Formation water chemistry is reflected in the indigenous microbial community composition

In producing wells where produced fluids contained <10% seawater (recently drilled wells and/or with long retention time), influence of the seawater injection appears to be negligible and the microorganisms identified could be considered primarily as indigenous to the oil field. In these producing wells, statistical analyses (RDA, Pearson correlations and hierarchical clustering) indicated that microbial community composition and metabolic potential was shaped by the produced water composition (Figures 2b and c), which is strongly influenced by the formation water composition. Therefore, this result suggests that formation water was a major factor affecting the composition and metabolic potential of the initial reservoir microbiome.

Archaeal community composition was similar in all these wells, with a predominance of thermophilic lineages (*Archaeoglobales* and *Thermococcales*), commonly observed in hot oil reservoirs (>70 °C) (Stetter and Huber, 2000; Gittel *et al.*, 2009). Metagenomic mining, construction of genomic bins and functional gene sequencing (*dsrAB*) indicated a strong potential for hydrogen sulfide production in these *Archaea*. Cultivated *Thermococcales* gain energy by fermentation using peptides as the carbon source and most of them require elemental sulfur as an electron acceptor (Gorlas *et al.*, 2015). Consistently, abundant sulfide dehydrogenase genes (*hydBG*) involved in elemental sulfur/polysulfide reduction in *Thermococcus* were identified. Capacity for hydrocarbon degradation coupled to sulfate reduction in members of the *Archaeoglobales* was also detected in the Halfdan oil field, supporting previous genomic analysis of the *Archaeoglobus* lineage (Khelifi *et al.*, 2014). The prevalence of methanogenic lineages (identified by 16S rRNA and *mcrA* genes sequencing) was dependent on the formation water associated with the sampled wells. Ninety percent (9/10) of the wells associated with FWA harbored detectable methanogens, whereas they were detected in <50% (9/19) of the wells associated with FWB. This suggests that formation water composition has an important influence on the occurrence of methanogens in petroleum reservoirs. Genomic bins of two methanogens affiliated to *Methermicoccus* and *Methanoculleus* indicated that H_2_ and CO_2_ were the predominant substrates for methanogenesis (Figure 4), which is consistent with the predominance of CO_2_ reducing methanogens in petroleum reservoirs, especially at high *in situ* temperature (Nilsen and Torsvik, 1996, Wentzel *et al.*, 2013). FWB had higher sulfate levels than FWA, and it is possible that the methanogens may be limited by competition with sulfate reducers for hydrogen (Oremland and Polcin, 1982) or inhibited by the elevated salinity in that particular oil accumulation (Waldron *et al.*, 2007).

The bacterial community composition was different in wells where the formation waters were considered ‘pristine’ compared with seawater-influenced wells. This confirmed the central role of formation water in structuring the indigenous microbial communities in the Halfdan field. In wells producing from the northern oil accumulation (FWA, moderate salinity, low sulfate and metal ions, Supplementary Figure S1, Supplementary Table S1), 16S rRNA gene sequencing indicated that members of the *Clostridia*, *Pelobacter* and *Thermovirga* were predominant (Figure 3). Genes involved in acetogenesis and degradation of a range of amino acids were identified in *Thermovirga* bins (Figure 4), which is consistent with known properties of cultured representatives of the genus *Thermovirga*. Indeed, *Thermovirga lienii*, which has been isolated from another hot oil reservoir in the North Sea, was shown to ferment proteinaceous substrates, amino acids and a limited range of organic acids, but not alkanes (Dahle and Birkeland, 2006). However, homologs of the benzylsuccinate synthase gene (*bssA*) for activation of alkyl-substituted aromatic hydrocarbons were detected in the most complete *Thermovirga* bin as well as in metagenomic reads (5.5±4% of all *bssA* genes detected), suggesting the possibility that *Thermovirga* spp. potentially have a role in aromatic hydrocarbons degradation. Similarly, genomic bins of *Clostridia* and *Pelobacter* highlighted considerable potential for peptide degradation and production of volatile fatty acids through fermentation. This is consistent with the significant correlations observed between relative abundance of 16S rRNA genes from *Clostridia* and *Pelobacter*, and propionate (Pearson correlation *r*>0.44, *P*<0.04), as well as acetate (Pearson correlation *r*>0.41, *P*<0.05) concentrations in produced fluids. Together this indicated that in low sulfate, and low-nutrient formation waters, indigenous microbial communities were driven by fermentation of complex carbon sources and anaerobic hydrocarbon degradation potentially linked to archaeal hydrogenotrophic methanogenesis as previously suggested by crude-oil biodegradation experiments (Jones *et al.*, 2008). Thus, not only did formation water composition affect the microbial community composition, but the microbial communities also influenced formation water composition by fermentation of organic carbon and generation of volatile fatty acids.

By contrast, the bacterial communities in ‘pristine’ wells from the second oil accumulation in the field (FWB: higher salinity, sulfate and metallic ions concentrations) were dominated by *Petrotoga* and *Desulfotomaculum* species. The metabolic potential of these communities was also distinct, with abundant genes involved in degradation of cellulose and other complex glycans (for example, laminarin and fucose), frequently found in thermophilic lineages (Blumer-Schuette *et al.*, 2008). Taxonomic assignment of these genes, as well as genomic binning identified members of the Thermotogales as the primary polysaccharide degraders (Figure 3). Genetic capacity for peptide and sugar degradation as well as fermentation was also detected in other genomic bins affiliated to minority lineages (Candidate division OP9, *Proteiniphilum, Draconibacterium*, *Brooklawnia*). These catabolic properties are commonly observed in thermophilic microorganisms isolated from oil reservoirs (Magot, 2005). Peptides and sugars are components of microbial biomass and marine detrital particles, suggesting that the bacterial food chain in petroleum reservoirs with higher salinity can also be supported by degradation of indigenous microbial necromass (Jørgensen and Marshall, 2016) or by the accumulation of marine organic matter in the formation water and sediments, originating from the marine shelf depositional system where the Halfdan field was formed (Albrechtsen *et al.*, 2001; Mardanov *et al.*, 2009). Interestingly, there is emerging evidence that deep biosphere communities utilize detrital carbohydrates and peptides as carbon and energy sources, which seem to be present in deep subsurface sediments (Lloyd *et al.*, 2013; Orsi *et al.*, 2013). Potential for hydrocarbon degradation (*bssA* gene) was detected in a genomic bin of a *Desulfotomaculum*-related bacterium, an observation consistent with previous survey of benzylsuccinate synthase genes (Callaghan *et al.*, 2010) and the occurrence of putative hydrocarbon-degrading *Desulfotomaculum* species in enrichments cultures (Tan *et al.*, 2015). The detection of these potential hydrocarbon-degrading anaerobic taxa in FWB suggests that even under relatively high salinity conditions oil biodegradation may occur in the reservoir.

These data indicated that petroleum reservoir formation water composition is an important factor shaping the potential activity and community composition of the indigenous microbes, which in turn gradually modify the formation water composition by production of metabolic products (for example, methane, volatile fatty acids or hydrogen sulfide). The low sulfate concentration in produced fluids, compared with formation water samples, from wells, which exhibit little influence of seawater injection (Supplementary Table S1), may be explained by activity of the indigenous sulfate reducers, identified by metagenomic sequencing, which could have been stimulated by alleviation of mass transfer limitation by fluid production.

### Oil production practices induce a systematic shift of reservoir microbial communities

With seawater injection for secondary oil production, the proportion of seawater in the oil reservoir usually increases progressively over the lifetime of an oil field, raising the threat of microbially induced reservoir souring. Dissimilatory sulfite reductase gene sequencing (Supplementary Figure S7), metagenome mining (Figure 3) and genomic bin reconstruction (Bins with *dsrAB* genes *n*=11; 9 *Deltaproteobacteria*, 1 *Desulfotomaculum*, 1 *Archaeoglobus*; Figure 4) identified a large diversity of potential hydrogen sulfide producers (1-D_Simpson_ *dsrAB* gene amplicons: 0.52±0.16), confirming that microbially driven souring is at least in part attributed to sulfate-reducing *Deltaproteobacteria,* or *Archaeoglobales*, as reported for other high temperature reservoirs (Gittel *et al.*, 2009). However, genes for other sulfidogenic processes were identified by metagenomic mining, indicating that souring potential is not limited to sulfate reducers. The potential for polysulfur reduction by members of the *Thermococcales* was identified, underlining the potential contribution that *Archaea* (*Archaeoglobales* and *Thermococcales*) may have in hydrogen sulfide production in high temperature petroleum reservoirs (Stetter and Huber, 2000). In addition, hydrogen sulfide can also be generated by non-enzymatic reaction when thiosulfate or elemental sulfur act as electron acceptors oxidizing hydrogen produced by fermentative organisms such as members of the *Thermotogales* (Takahata *et al.*, 2001) that were identified in the Halfdan system.

As a mitigation strategy for microbial reservoir souring in Halfdan, seawater is continuously enriched with 100–150 mg l^−1^ of nitrate before injection. Nitrite and nitrate were only detected in producing wells with seawater breakthrough (seawater proportion above 50%) and the effect of nitrate dosing for souring mitigation was evident in the microbial communities, as previously reported (Gittel *et al.*, 2012). A probable consequence of this anthropogenic alteration of the oil field redox potential is the lower representation of sulfate reduction genes (*dsrAB*) and sequences from sulfate-reducing microorganisms in 16S rRNA gene libraries from samples influenced by seawater injection. By contrast, abundant 16S rRNA and nitrate reduction genes recovered from members of the *Deferribacteres* lineages were detected, indicating a major role for *Deferribacteres* in nitrate reduction in these seawater-rich produced fluids (Figure 3) (Gittel *et al.*, 2012). *Deferribacteres* species described so far are moderate thermophiles (optimal temperature 60 °C) and can utilize nitrate, iron and elemental sulfur as electron acceptors (Green *et al.*, 1997; Takai *et al.*, 2003; Slobodkina *et al.*, 2009), which is consistent with the genetic potential detected in the *Deferribacteres* bin. Potential for anaerobic aromatic compound degradation was also present in a *Deferribatceres* bin recovered from a seawater-influenced well. Capacity for aromatic compound degradation by cultivated *Deferribacteres* has never been tested but this catabolic ability may explain the rapid colonization of organisms from the *Deferribacteres* in nitrate-treated offshore oil reservoirs (Magot *et al.*, 2000; Lysnes *et al.*, 2009; Gittel *et al.*, 2012). Together, these results provide field-based evidence that nitrate injection for souring control successfully limited the development of sulfate-reducing microorganisms by creating an environment conducive for a second generation of moderately thermophilic microorganisms with metabolic capacities centered on hydrocarbon degradation coupled to nitrate reduction. Furthermore, the nitrite accumulation observed in the wells is consistent with reports of incomplete nitrate reduction to nitrite in oil fields with temperature above 50 °C (Fida *et al.*, 2016). Interestingly, this shift in microbial community composition was observed in wells producing oil associated with both FWA and FWB, as well as in other high temperature oil fields (Magot *et al.*, 2000; Lysnes *et al.*, 2009; Gittel *et al.*, 2012). This suggests that the selection pressure generated by the secondary production strategy overcomes the indigenous communities irrespective of the formation water composition or the nature of the native reservoir community.

### A third-generation microbiome?

In producing wells with the longest history of seawater exposure (>12 years in service as of 2014), produced fluids, comprised >95% seawater, leading to a decrease of the *in situ* temperature (~40 °C). These very long-term seawater-injected producing wells exhibit a further shift in microbial community composition. Consistent with the cooling of the reservoir, the relative abundance of thermophilic *Archaea* decreases whereas mesophilic *Epsilon*- and *Gammaproteobacteria*, previously identified in the seawater and injected fluids of this and other oil facilities (Hubert and Voordouw, 2007; Gittel *et al.*, 2012), were detected in higher abundance by qPCR, 16S rRNA gene sequencing and metagenomic mining. Reconstructed genomes from organisms likely to be exogenous to the field displayed considerable metabolic versatility with the capacity to use a large variety of substrates for energy generation (sulfide oxidation coupled to nitrate or nitrite reduction or denitrification for *Epsilonproteobacteria*;, sulfide and sulfur oxidation, thiosulfate reduction, methane and methanol oxidation, and nitrate or nitrite reduction for *Gammaproteobacteria* lineages). Substrates to support the metabolic repertoire of these microorganisms were presumably furnished by the large amount of injected seawater and/or produced by the microbial communities that were dominant during the earlier stages of the reservoir’s production history (for example, methane, volatile fatty acids or hydrogen sulfide).

Together these results showed that the new redox and temperature conditions generated by extended secondary production involving nitrate injection provide a new environment favorable for a third-generation of fast growing, versatile and mesophilic seawater microorganisms that outcompete slowly growing indigenous thermophilic microbes, as well as members of the *Deferribacteres*, which were prevalent in higher temperature wells and when lower levels of seawater breakthrough were apparent. However, since the rapid growth of exogenous microorganisms may lead to plugging of porosity in the reservoir, and the elemental sulfur generated by sulfide oxidation is as corrosive as hydrogen sulfide (Little *et al.*, 2000), the shift of the microbial community composition to fast growing microbes with energetically efficient metabolisms, boosted by the increase in mass transfer induced by fluid movement during oil production, could have significant practical consequences for the oil industry.

## Conclusion

Produced fluids provide a window on the petroleum reservoir biosphere. Therefore, monitoring produced water microbial community composition and metabolic potential is essential for an accurate evaluation of the potential for development of production associated problems and development of an effective oil field microbial control program. Our investigation of the Halfdan oil field, exposed to a gradient of conditions driven by oil production practices over the lifetime of the field, provides novel information about the microorganisms and their functions across the space and lifetime of an offshore oil field. First, this study demonstrated that a large variability of the microbial community composition and metabolic profile occurs across the different producing wells of a single oil field. Consequently, microbial community analysis of a single well is not representative of an entire oil field microbiome, and extended investigation is mandatory to explore the health of an oil field. Second, analysis of producing wells at their primary production stage revealed the important role of the formation water composition (salinity, sulfate and other ions concentration) in the make-up of indigenous reservoir microbial communities and that multiple native reservoir communities can co-occur in a single oil reservoir when different oil accumulations are present. Third, our results provided evidence that anthropogenic interventions for oil production are significant drivers of the oil field microbiome and engender considerable spatial and temporal variability of oil field microbial communities regardless of the indigenous microbiome. When secondary production begins, injected fluids cool the reservoir and deliver exogenous oxidants, changing the microbial communities considerably as the energetic potential of the environment increases over the production lifetime of the field. Anthropogenic perturbation of the oil field ecosystem may appear to effectively limit the souring capacity of the oil reservoir microbiome. However, extended water flooding may open new avenues to metal corrosion and plugging when the temperature becomes permissive and the proportion of exogenous bacteria outcompete the indigenous communities. Microbial monitoring and control programs therefore, should account for a diverse and dynamic system ensuring adequate sampling coverage and frequency.
