# Supplementary material for: Succession in the petroleum reservoir microbiome through an oil field production lifecycle
Source: ISME J. 2017 May 19;11(9):2141–54. doi: 10.1038/ismej.2017.78 (PMC5563965; doi:10.1038/ismej.2017.78)
Supplement: Supplementary Material [file ismej201778x1.docx]

**Materials and Methods**

DNA extraction

Nucleic acids were extracted in a sterile hood using a FastDNA Kit for Soil (MP Biomedicals, Santa Ana, CA, USA) with the following modifications. Filters were aseptically cut in approximatively 1 cm^2^ pieces then placed in two Lysing matrix tubes with 200 µg of Poly A-salt (MP Biomedicals, Santa Ana, CA, USA) for independent duplicate DNA extractions. Furthermore, bead-beating step (30s at 5.5m/s) was repeated three times and lysing matrix tubes with samples were frozen in liquid nitrogen and thawed at 56°C between each bead-beating step. The centrifugation step after protein precipitation was repeated twice to limit contamination of the DNA with enzyme inhibitors.

qPCR

Amplification reactions were performed in triplicate with a Rotor-Gene Q system (Qiagen, Hilden, Germany) in a final volume of 25 µl using Brilliant III master Mix (Agilent Technologies, Santa Clara, CA, USA), 0.5 µM of each primers and 0.1 ng of DNA template. qPCR conditions were as follows: 40 cycles of denaturation at 95°C for 15 s then annealing at an appropriate temperature for 30 s and extension at 60°C for 30 s. Standard curves were prepared in triplicate with dilutions ranging from 0.001 to 100 nM of DNA extracted from *Desulfobulbus propionicus* (ATCC 33891) and *Methanococcoides methylutens* (ATCC 33938). The R^2^ of standard curves obtained by real-time PCR were above 0.997 and PCR efficiencies were above 92% and 94% for *Archaea* and *Bacteria* respectively.

Abundances of the *Epsilonproteobacteria* and *Deferribacteres/Flexistipes* was estimated using EPS-F /EPS-R and DFB-F / DFB-R primer sets respectively while abundances of *Achaeoglogales* and *Thermococcales* were investigated using 242F-ArchaeoG / 438R-ArchaeoG and 993F-Thermo / 1063R-Thermo primer sets respectively (Supplementary Table S2). Standard curves for each quantification were generated using dilutions of genomic DNA from pure cultures of *Sulfurospirillum multivorans* DSM12446 (*Epsilonproteobacteria*), *Flexistipes sinusarabici* DSM4947 (*Deferribacteres-Flexistipes*), *Thermococcus zilligii* DSM2770 (*Thermococcales*) and *Archaeoglobus fulgidus* DSM4304 (*Archaeoglobales*). The R^2^ values of the standard curves were above 0.996 and PCR efficiencies above 90% for all reactions. As with quantification of *Bacteria* and *Archaea*, 1 ng, 0.5 ng and 0.1 ng of DNA were used as qPCR template.

Library Preparation and Sequencing

16S rRNA genes were amplified in duplicate from two independent DNA extracts (* and ¤) using Brilliant III master Mix (Agilent Technologies, Santa Clara, CA, USA), 500 nM of each primer fused with Illumina adaptors (Supplementary Table S2) and 1 ng of DNA template on 25µl reaction volume. PCR conditions were as follows: denaturation at 95°C for 15 s then annealing at 58°C for 30 s and extension at 72°C for 30 s. To detect potential overestimation of specific lineages due to preferential amplification during the reaction, the DNA extracts (*) were amplified with 30 cycles of amplification whereas DNA extracts (¤) were amplified with only 20 cycles. No significant difference of taxonomic affiliation of the amplicons were observed (P>0.4) between each of the replicates.

*mcrA* and *dsrAB* genes were amplified in triplicate from a single DNA extract per sample using Brilliant III master Mix (Agilent Technologies, Santa Clara, CA, USA), 500 nM of each primer fused with Illumina adaptors (Supplementary Table 2) and 1 ng of DNA template in a 25µl reaction volume. The PCR program used was the same as for 16S rRNA gene with an adapted annealing temperature (Supplementary Table S2)

Single Gene Analysis

MiSeq runs were demuliplexed and adapter plus barcode sequences were trimmed on instrument using Illumina’s MiSeq Reporter software. Fastq files were downloaded from the instrument and further quality filtered using Sickle (Joshi, 2011), with a Q-score cut-off of Q>20 and minimum read length of 180bp. Paired-end joining was done using the ‘join_paired_ends.py’ script bundled with the QIIME package (version 1.9) (Caporaso *et al.*, 2010), using default settings. Only reads which passed QF and successfully formed a paired-end joined read where used in further analyses. OTU picking and taxonomic assignment for gene analyses were done with QIIME using the ‘pick_open_reference_otus.py’ script and gene-specific databases. The Silva database (version 1.19) (Quast *et al.*, 2013) was used for 16S rRNA gene OTU picking and taxonomic assignments, *dsrAB* gene OTUs and taxonomic assignments were done against publicly available database (Muller *et al.*, 2015) and *mcrA* gene OTUs and taxonomic assignments were done against an in-house database.

Metagenome Data Analysis

Barcode and adapter sequences were trimmed from the metagenome datasets on instrument using Illumina’s MiSeq Reporter software and downloaded as fastq files. Datasets were quality filtered using Sickle (Joshi and Fass, 2011), using a Q-score cut-off of Q>20 and minimum read length of 60bp. Paired-end joining was done using the ‘join_paired_ends.py’ script bundled with the QIIME package (version 1.9), using default settings. Joined and unjoined (due to longer insert size and/or one read direction not passing quality filtering) read files were concatenated into a single fastq file. Metagenomic assembly was done on the single merged file using MEGAHIT (Li *et al.*, 2015), using default settings. After assembly, all reads which passed quality filtering where mapped back to the assembled contigs to detect reads which were not included in the assembly using BBMap (Bushnell, 2014), using default settings. Reads which were not mapped to the assembly were concatenated with assembled contigs into a single fasta file for upload to the IMG/M analysis pipeline (Markowitz *et al.*, 2008) for gene calling and functional annotation.

Draft Genome Analysis:

To generate longer contigs and allow for the highest likelihood of producing near-complete draft genomes, the reads from all eight metagenomes were concatenated. The concatenated fastq files were quality filtered using Sickle (Joshi and Fass, 2011) (Q>20; length>60bp). Only reads which passed quality filtering in both directions (R1 and R2) where kept. Forward and reverse (R1 and R2) quality filtered read files were interleaved and converted to fasta format using the ‘fq2fa’ program bundled with the IDBA-UD assembler (Peng *et al.*, 2012). Due to the size of the fasta file generated through the combination of all metagenomes digital normalization was required. Normalization was done through the reduction of redundant reads using the ‘filter-adund.py’ script within the khmer package (version 1.4.1) (Crusoe *et al.*, 2015) using a kmer length of 32 and maximum kmer abundance of 5. The normalized fasta file was assembled using IDBA-UD (Peng *et al.*, 2012) using default settings. Assembled contigs were uploaded to IMG for gene calling and functional gene annotation. The combined metagenome assembly is available from IMG under accession number 3300005882. Draft genome binning was done using all contigs over 5,000bp in length with MetaBAT (Kang *et al.*, 2015), using default settings. Resulting bins were analyzed for quality and completeness against single copy genes using CheckM (Parks *et al.*, 2015). Bins with completeness above 50% and contamination level under 3% were selected for further analysis. Metabolic pathways were considered as present in the assembly if more than 75% of the pathways were detected using Metacyc and Kegg pathway mapping tool. Additionally, key genes for functional pathways were also manually screened to validate the presence of the pathway in the draft genomes. For *mcrA*, *dsrAB*, *pmoA*, *assA*/*bssA*/*masD*, *mtrAB*/*omcAB* genes analysis, contigs were blasted against publicly available databases.

**References**

Bushnell B. (2014). BBMap: A Fast, Accurate, Splice-Aware Aligner. Ernest Orlando Lawrence Berkeley National Laboratory, Berkeley, CA (US).

Caporaso JG, Kuczynski J, Stombaugh J, Bittinger K, Bushman FD, Costello EK, *et al.* (2010). QIIME allows analysis of high-throughput community sequencing data. *Nat Methods* **7**: 335–336.

Crusoe MR, Alameldin HF, Awad S, Boucher E, Caldwell A, Cartwright R, *et al.* (2015). The khmer software package: enabling efficient nucleotide sequence analysis. *F1000Research* **4**: 900.

DeLong EF. (1992). Archaea in coastal marine environments. *Proc Natl Acad Sci* **89**: 5685–5689.

Gittel A, Kofoed MVW, Sørensen KB, Ingvorsen K, Schramm A. (2012). Succession of Deferribacteres and Epsilonproteobacteria through a nitrate-treated high-temperature oil production facility. *Syst Appl Microbiol* **35**: 165–174.

Joshi N, Fass J. (2011). Sickle: A sliding-window, adaptive, quality-based trimming tool for FastQ files (Version 1.33)[Software]. *Available HttpslégitbubcoménajoshiéchkleQOll*.

Kang DD, Froula J, Egan R, Wang Z. (2015). MetaBAT, an efficient tool for accurately reconstructing single genomes from complex microbial communities. *PeerJ* **3**: e1165.

Klindworth A, Pruesse E, Schweer T, Peplies J, Quast C, Horn M, *et al.* (2013). Evaluation of general 16S ribosomal RNA gene PCR primers for classical and next-generation sequencing-based diversity studies. *Nucleic Acids Res* **41**: e1–e1.

Li D, Liu C-M, Luo R, Sadakane K, Lam T-W. (2015). MEGAHIT: An ultra-fast single-node solution for large and complex metagenomics assembly via succinct de Bruijn graph. *Bioinformatics*. e-pub ahead of print, doi: 10.1093/bioinformatics/btv033.

Lipp JS, Morono Y, Inagaki F, Hinrichs K-U. (2008). Significant contribution of Archaea to extant biomass in marine subsurface sediments. *Nature* **454**: 991–994.

Luton PE, Wayne JM, Sharp RJ, Riley PW. (2002). The mcrA gene as an alternative to 16S rRNA in the phylogenetic analysis of methanogen populations in landfillb. *Microbiology* **148**: 3521–3530.

Markowitz VM, Ivanova NN, Szeto E, Palaniappan K, Chu K, Dalevi D, *et al.* (2008). IMG/M: a data management and analysis system for metagenomes. *Nucleic Acids Res* **36**: D534–D538.

Muller AL, Kjeldsen KU, Rattei T, Pester M, Loy A. (2015). Phylogenetic and environmental diversity of DsrAB-type dissimilatory (bi)sulfite reductases. *ISME J* **9**: 1152–1165.

Parks DH, Imelfort M, Skennerton CT, Hugenholtz P, Tyson GW. (2015). CheckM: assessing the quality of microbial genomes recovered from isolates, single cells, and metagenomes. *Genome Res* **25**: 1043–1055.

Peng Y, Leung HCM, Yiu SM, Chin FYL. (2012). IDBA-UD: a de novo assembler for single-cell and metagenomic sequencing data with highly uneven depth. *Bioinformatics* **28**: 1420–1428.

Quast C, Pruesse E, Yilmaz P, Gerken J, Schweer T, Yarza P, *et al.* (2013). The SILVA ribosomal RNA gene database project: improved data processing and web-based tools. *Nucleic Acids Res* **41**: D590–D596.

Rutherford A. (2014). Abundance and Distribution of Major and Understudied Archaeal Lineages at Globally Distributed Deep-Sea Hydrothermal Vents. *Diss Theses*. e-pub ahead of print, doi: 10.15760/etd.1554.

Stagars MH, Ruff SE, Amann R, Knittel K. (2015). High Diversity of Anaerobic Alkane-Degrading Microbial Communities in Marine Seep Sediments Based on (1-methylalkyl)succinate Synthase Genes. *Front Microbiol* **6**: 1511.

Suzuki MT, Taylor LT, DeLong EF. (2000). Quantitative Analysis of Small-Subunit rRNA Genes in Mixed Microbial Populations via 5′-Nuclease Assays. *Appl Environ Microbiol* **66**: 4605–4614.

Yu Y, Lee C, Kim J, Hwang S. (2005). Group-specific primer and probe sets to detect methanogenic communities using quantitative real-time polymerase chain reaction. *Biotechnol Bioeng* **89**: 670–679.

**Supplementary Data**

- Table S1: Geochemical parameters from each producing well and composition of the formation waters. All data were measured at the same date as microbial sampling (Dec. 2014), excepted nitrate concentration (data from 2013). NA: not available; PW: produced Water; FW: Formation Water. Compl.: completion; Temp.: Temperature; Salinity was determined from chloride concentration.


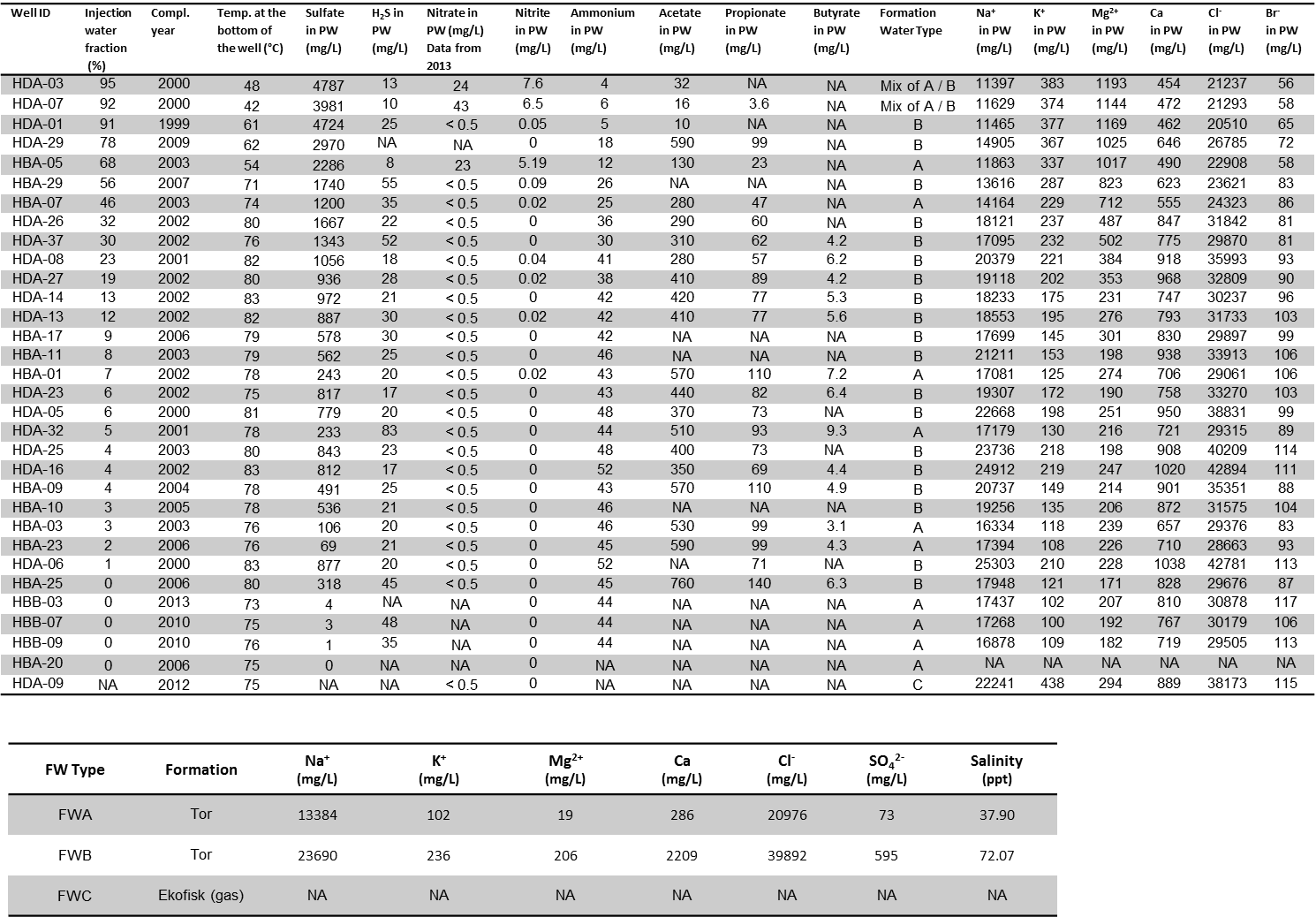


-
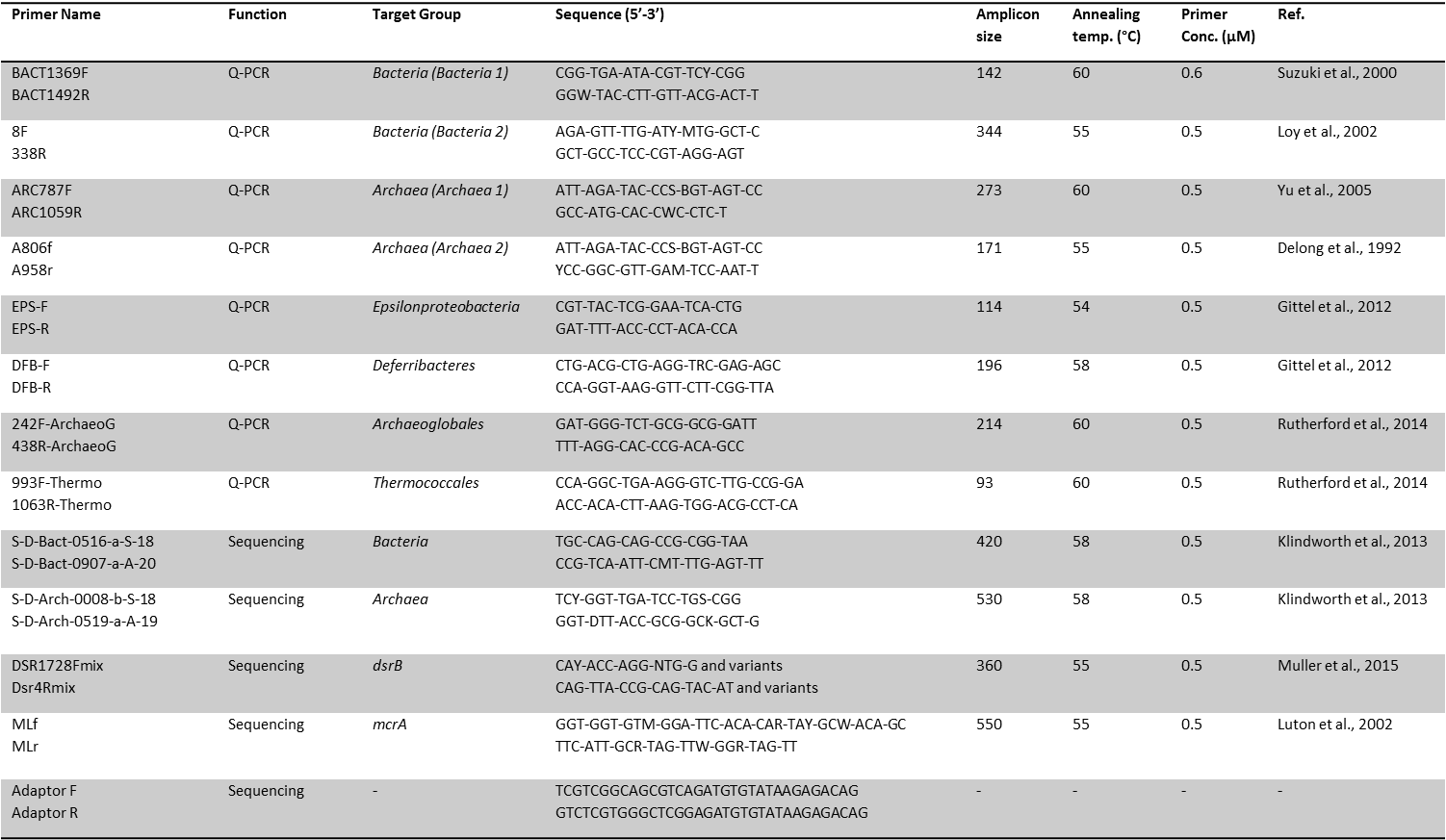
Table S2: List of primer sets used for this study
- Figure S1: Schematic map of the Halfdan Oil field.

- Figure S2: Taxonomic affiliation of the archaeal 16S rRNA gene amplicons for all produced fluid samples according to the main geochemical parameters of the wells. DHVEG-1: Deep-sea Hydrothermal Vent Euryarchaeotal Group 1


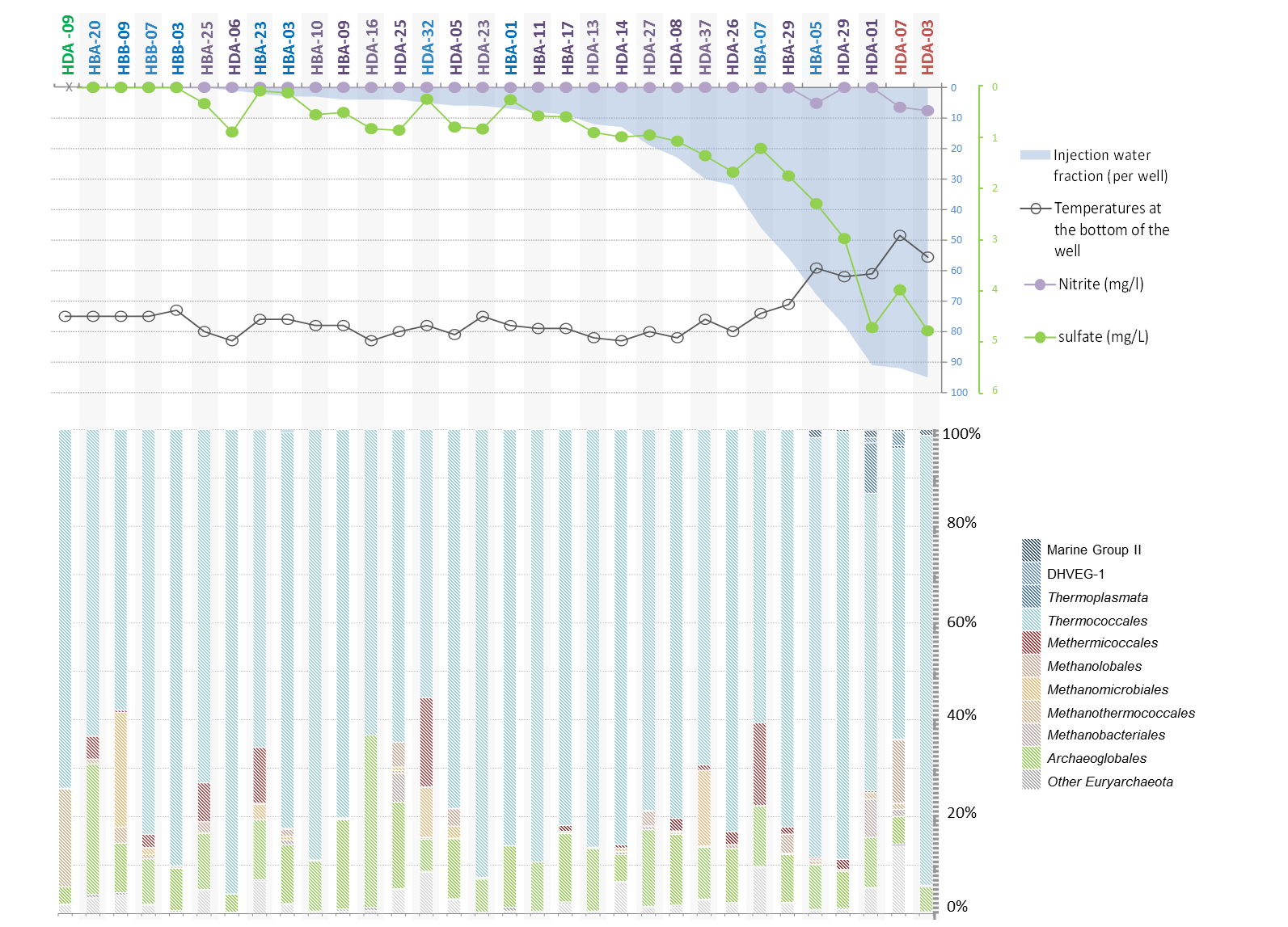


- Figure S3: Taxonomic affiliation of the 16S rRNA gene amplicons (replicate * and ¤) and metagenomic 16S rRNA genes (MG) for a) Bacteria and b) Archaea in produced fluid samples selected for metagenomic analysis.

- Figure S4: Comparison of the relative proportion of the 16S rRNA gene amplicons and metagenomic 16S rRNA genes affiliation for a) Bacteria and b) Archaea in all producing fluid samples.

**
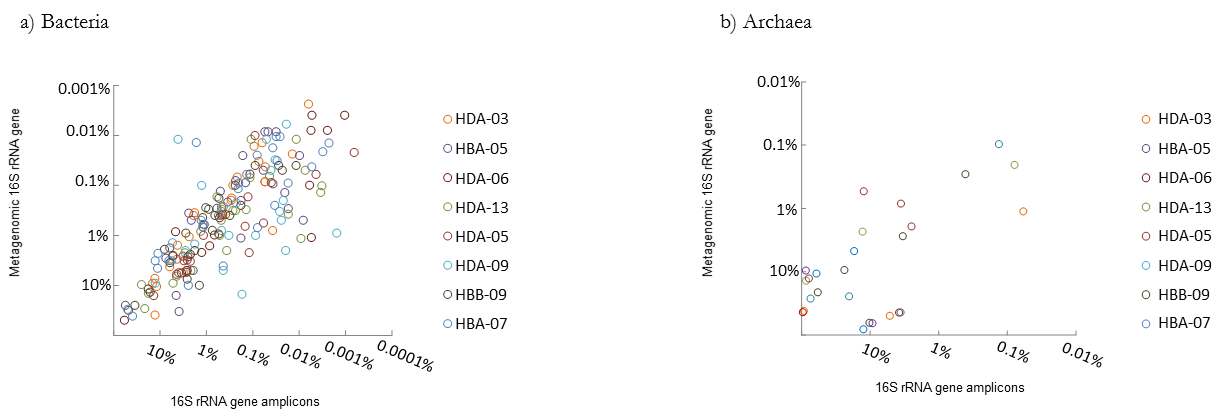
**

- Figure S5: 16S rRNA gene abundance per milliliter of produced fluid. a) *Bacteria*, *Deferribacteres* and *Epsilonproteobacteria* 16S rRNA gene abundance. b) *Archaea*, *Thermococcales* and *Archaeoglobales* 16S rRNA gene abundance.


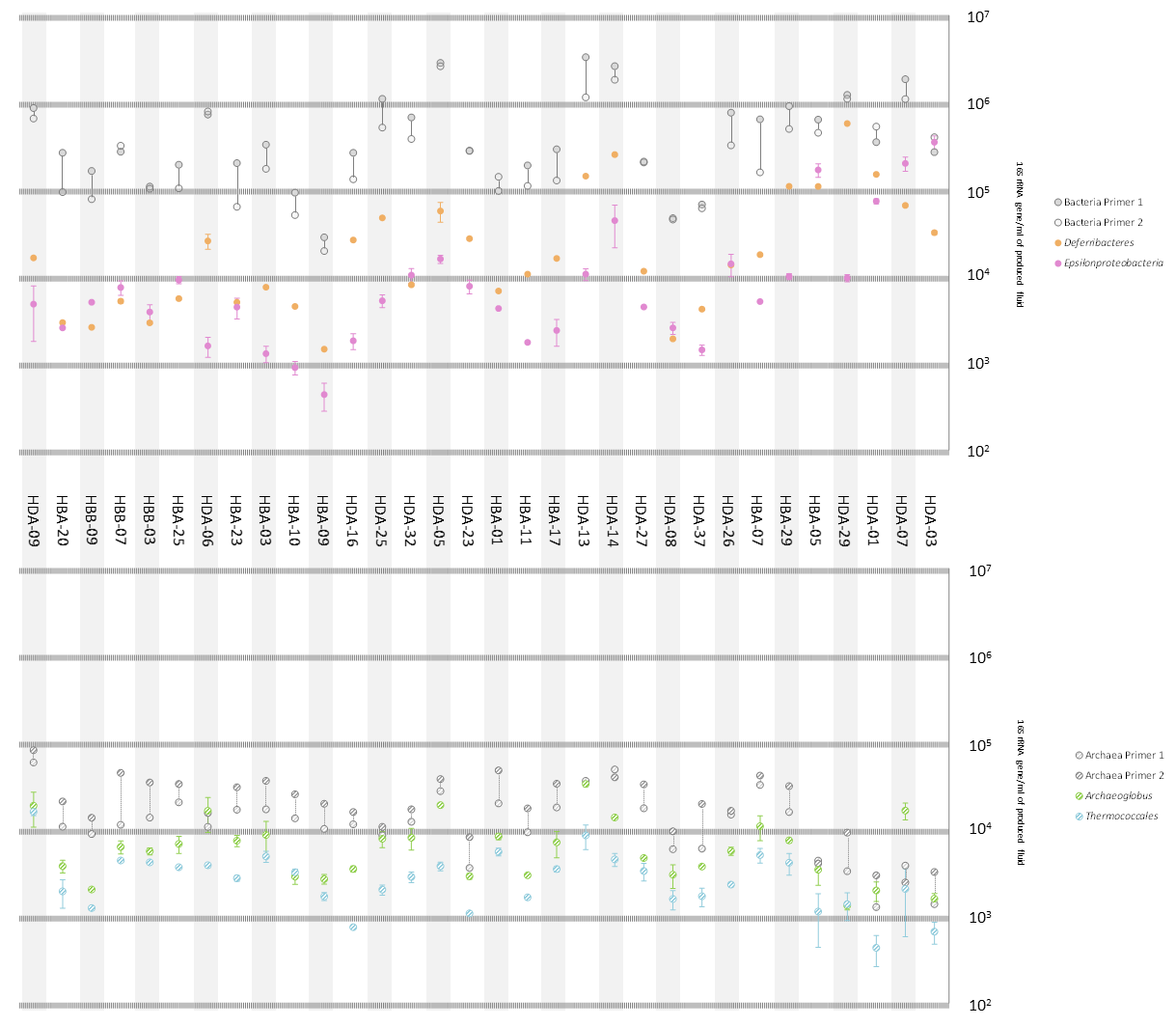


-
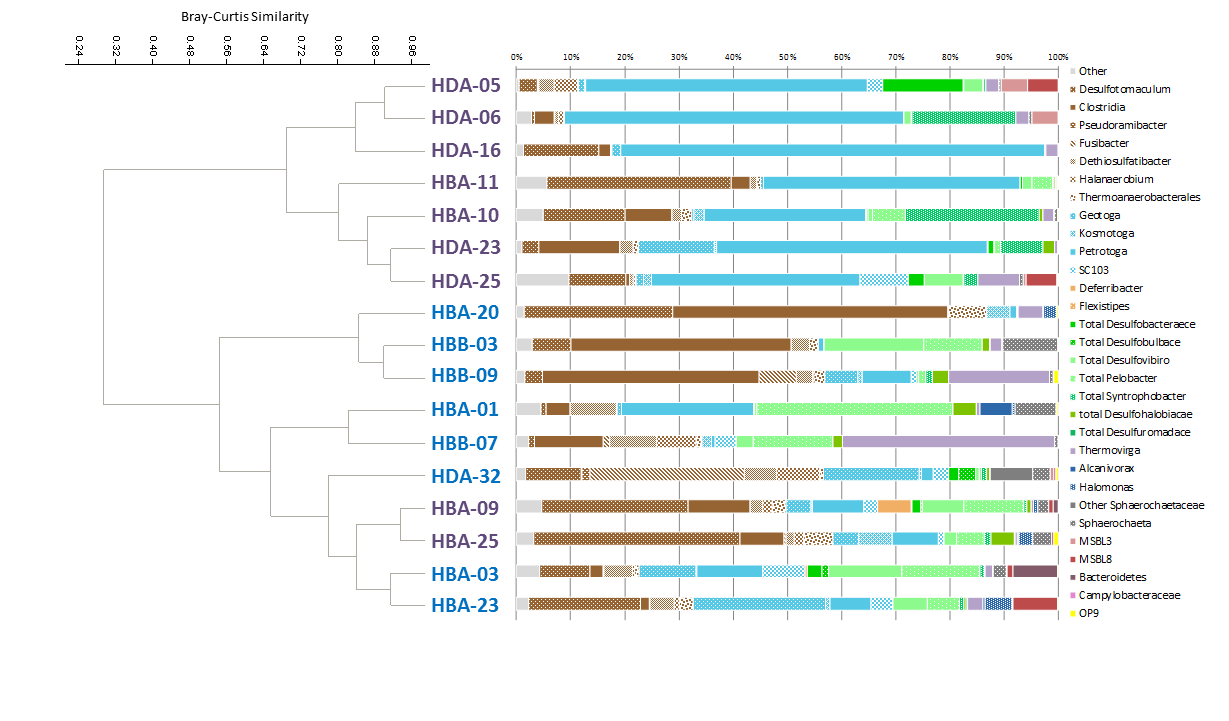
Figure S6: Taxonomic affiliation of the bacterial 16S rRNA gene amplicons for wells with produced fluid with less than 10% of seawater. Clustering is based on Bray-Curtis similarity measure. Samples labeled in Blue are from FWA and samples in Purple are from FWB.
- Figure S7: Taxonomic affiliation of dissimilatory sulfite reductase gene (*dsrAB*) amplicons for all produced fluid samples according to the main geochemical parameters of the wells.


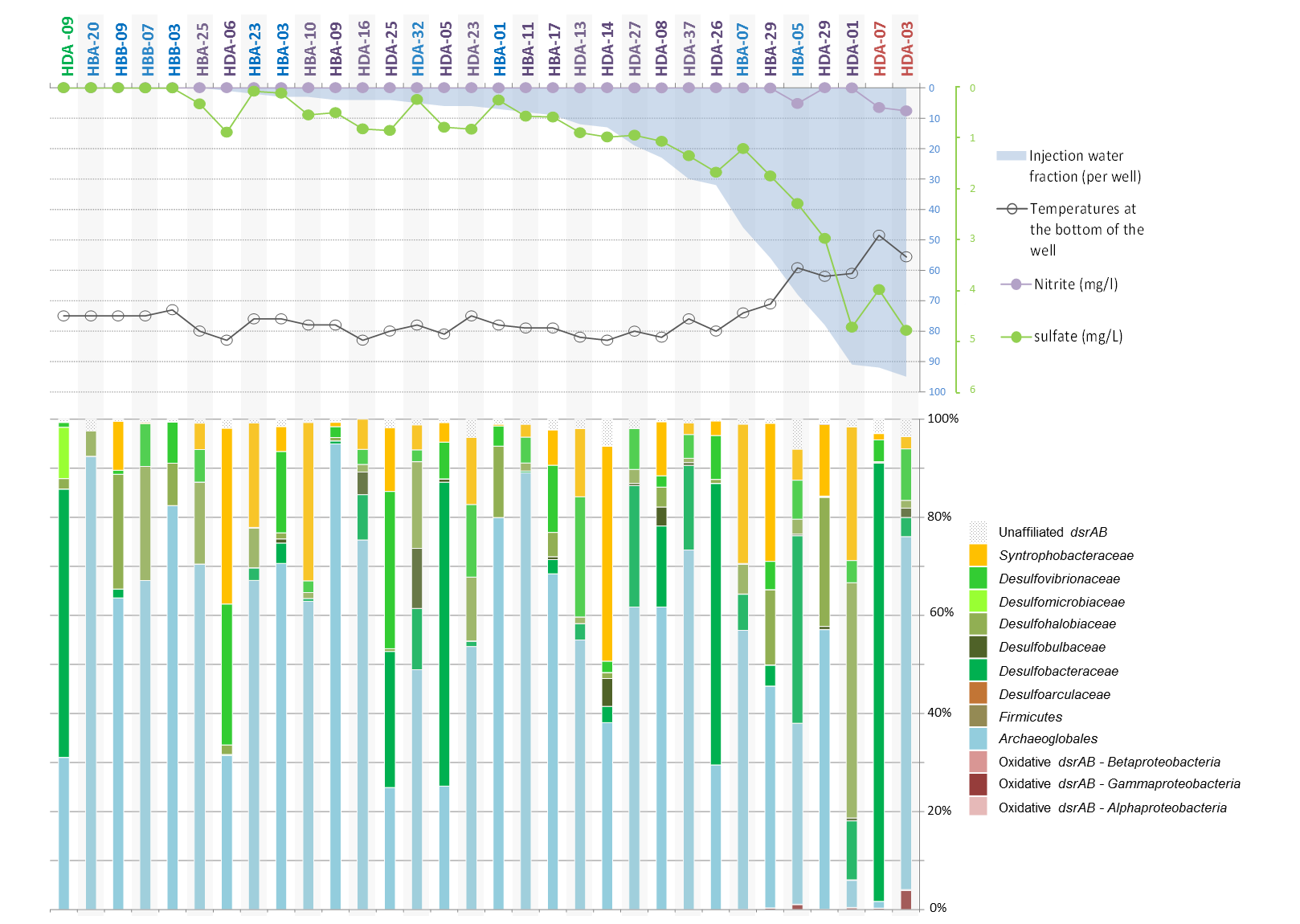

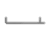


X

-
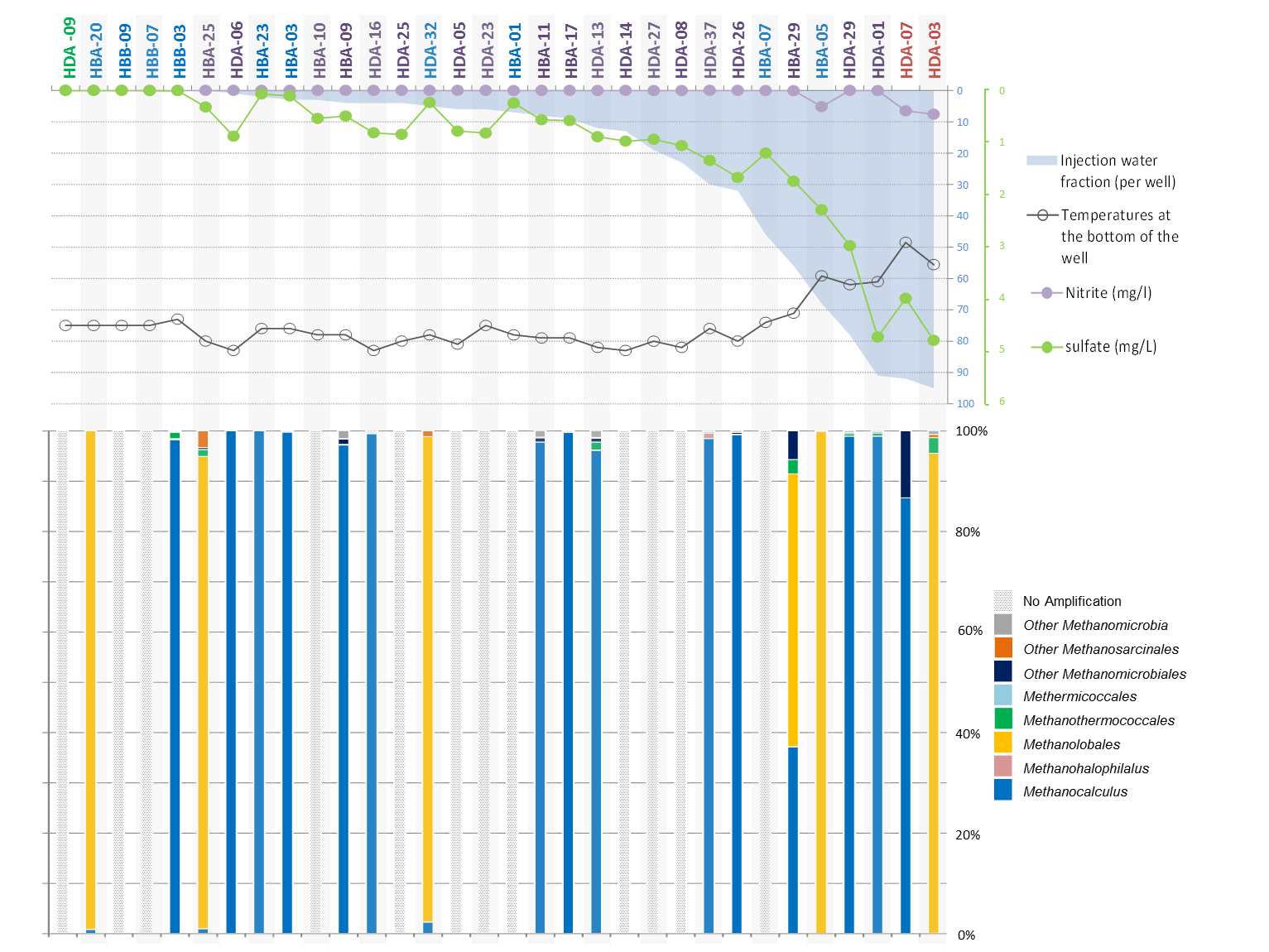
Figure S8: Taxonomic affiliation of the methyl coenzyme M reductase A (*mcrA*) amplicons for all produced fluid samples according to the main geochemical parameters of the wells.


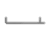


X
